# New Employers' Subscription Scheme in Detail

**Published:** 1920-02-07

**Authors:** 


### AN EXAMPLE OF LEADERSHIP AT LEEDS.

### New Employers' Subscription Scheme in Detail.

Further evidence of the rally to the Voluntary Hospital System is forthcoming, and, since ideas do more, perhaps, than enthusiasm to achieve results, our readers will be interested in the following particulars of a scheme just launched at Leeds. The object is to raise the permanent income of the Leeds General Infirmary. The author of the idea is Mr. T. F. Braime, and on his suggestion a letter has been addressed to over fifty of the largest firms in Leeds, which have endorsed the suggestion contained within it. That suggestion, briefly, is to give to the General Infirmary a subscription based upon a gift of 2s. per head per annum for every employee on their staffs. More than this, a few firms have suggested that the *pro rata* payment might be 3s., or even 4s., per head, in place of the smaller figure proposed by Mr. Braime. The plan has gone so well that the committee of the Leeds General Infirmary hopes eventually to bring nearly every firm in the city into co-operation; and when that shall have been done Mr. F. J. Bray, the general manager, will be instructed to approach the firms in the outlying districts to see if they cannot organise themselves in the same way. The root idea of the plan is to put appeals in their proper place, which is the place of emergency, and to secure an assured annual income. Such an income will be far more valuable, of course, than spasmodic but incessant appeals, which waste an immense amount of effort, achieve no final satisfaction, and are hardly less a worry to the public than to the hospital staff by which they have to be thought out and arranged.

The value of the idea, however, depends as much upon the method of its application as upon the principle whereon it is based. For this reason we go into some detail, and place on record the steps whereby it has been made a success. Mr. T. F. Braime's original letter ran as follows:—

Sirs,—As a member of the Board of the Leeds Infirmary Special Finance Committee, I take the liberty of asking for a kindly expression of your opinion upon a scheme I put before the Board a few weeks ago, to try and extricate the Infirmary from its chronic condition of indebtedness and the efforts which continually have to be made to keep it going by passing the hat round.

The position is a deplorable one, the expenditure—owing to the high price of everything—being slightly over £25,000 per annum more than the income. During the war £58,000 worth of negotiable securities have had to be sold to keep going. Mr. Watson's splendid gift will help to redeem some of these, unless the money is used to clear off the overdraft at the Bank (about £40,000).

The position now is that employers of labour in 1918 subscribed about £1,500—roughly 25 per cent.—towards the £6,190 13s. shown under the first item on the enclosed balance sheet. *There is no settled amount for each firm*, some firms employing only a small number giving, say, £10 to £20, and others employing large numbers giving £1 to £5, and, in a very large degree, nothing at all. The idea is to try and bring every firm in all trades into this scheme, so that every firm may contribute in equity and bear its fair share in supporting these splendid institutions, so that they may be relieved of the harassing thought of where the next week's wages and expenses are to come from, it being absolutely necessary the Infirmary should have a sure and reliable portion of its income that it can depend upon instead of relying on the spasmodic and uncertain gifts, donations, collections, etc., as at present.

In many cases firms are already subscribing as much as they would under this scheme, and in other cases more: it is from those who are now giving only small amounts and those who are not giving anything at all from whom the new income is expected to be obtained, and, in addition, *through the good example shown by employers, it is hoped to widen the list of personal subscribers who are not employers of labour*, and thus obtain from this source a further several thousands per year towards the general fund.

I enclose a rough outline of the scheme as proposed, which so far has met with the approval of every firm to whom it has been explained. The great thing the Board are anxious to know is, if you approve of the scheme, *do you consider that under the abnormally prosperous circumstances expected to rule during the next few years, that firms would be willing to contribute* 4s. *per employee per annum*—which would be 1d. per week per employee—instead of the 2s. or ½d. per week which I have named? If this could be done for only a few years it would place the institution upon a sound financial basis and enable it to deal with the larger number of patients—approximately 600 to 1,000 always waiting admittance—many of whom must die owing to the impossibility of treating these persons under present conditions. A frank expression of your opinion and as to whether you would support the scheme if it is decided to launch it would be greatly appreciated. As I have to make my report for next Friday's meeting, I should esteem a reply before that date if you can manage it, for which I thank you in anticipation.

Yours faithfully,

T. F. Braime.

We next set forth the details of the scheme itself, and place these on record by way of illustration and example, for an ill-understood detail is often sufficient to spoil the success of an excellent idea.

The scheme is called the Leeds General Infirmary Proposed Employers' Contribution Fund, and is comprised under four main heads, as follows:—

It is suggested that the principle or scheme for inaugurating the Fund should be advertised and brought to the notice of employers of labour somewhat on the following lines:—

1. That the Fund should be known by some such title as "The Leeds Infirmary Employers' Contributory Fund."

2. That in appeals by advertisement or direct to employers to give their support to this Fund, *the amount so raised shall be treated as separate from the general subscription list*, and *be shown as such in the accounts* of the Infirmary from time to time.

(The reason why it would be advisable to publish the employers' contributions separate from the others is, it would create a good impression amongst the workers or employees and the general public, showing that employers of labour recognise their responsibility towards those

**An Example of Leadership at Leeds**—(*continued*).

who run the risk of injury and ill-health whilst in their service, and especially as they would realise how this money is being given by employers of labour for the general good, and without hope on the part of the employers of any direct return excepting perhaps the gratitude of those who receive benefit from the institution. It would also have its influence upon other members of the community and perhaps induce them to become regular—or more liberal—subscribers to the general fund.)

3. It is suggested that the contribution by the employer should be at the rate of 2s. per employee per annum, *based on the average number of employees in each year,* to be applied to all trades equally.

4. That the payment of the contribution *be half-yearly,* say in January and July.

That the Fund be retrospective to July last, *on the distinct understanding that any firm who have paid a subscription in that period should have the amount of such subscription deducted from the amount they would pay under this scheme,* the reason for making the contribution retrospective being the present vital and urgent necessity of financial assistance being rendered at once, and the opportunity it gives to those who have not previously subscribed, or who have not adequately subscribed, to make good.

The suggested sum of 2s. per employee would be a perfectly equitable and reasonable amount for the employer to pay, and, moreover, a sum which is easily calculated. *It is far better to have a large number contributing willingly than a smaller number paying grudgingly,* and there is much greater prospect of getting all to join if the amount is a reasonable one. It should be borne in mind that *trade is likely to be better for many years to come, and that,* in consequence, *the larger the number of employed the larger the fund would become.*

Presuming the number of employees for Leeds to be 100,000 and 50,000 for the district around Leeds, or, say, 150,000 at 2s. each (and which I feel sure would be greatly exceeded) it would give a certain income from that source of £15,000, less, say, £2,500, the approximate amount to be deducted as *now being subscribed by employers* to the General Fund, or, say, a net gain of £12,500 per annum. Supposing £2,500 of this sum was allocated to the Leeds Public Dispensary, the Women and Children's Hospital, Leeds District Nursing Association, and the Maternity Hospital, £10,000 would be left, which would all be absolutely new income. *The amount to be set aside for these other institutions,* and the proportion of such sum to be allocated to each, *to be settled by three gentlemen independent of the institutions,* viz., the Lord Mayor (Mr. T. B. Duncan), the Stipendiary Magistrate (Mr. C. M. Atkinson), and the Town Clerk (Sir Robert Fox). *Should the Workpeople's Hospital Fund,* through its doubled or trebled subscriptions, *be able to give to the Infirmary £20,000 instead of* £10,000 *annually,* it would then *only remain for private subscriptions and donations* to be increased by, say, £5,000 per annum from all sources and the present annual deficit of £25,000 would be wiped out.

It is hoped this scheme may be successful. Its great and most valuable features is the probability of securing a permanent and reliable income in place of the present spasmodic and uncertain method of depending upon irregular gifts. *Every employer would know exactly what his proportion for the year would be,* and that if his neighbour employed double the number of men he would be paying his just proportion—neither more nor less.

*The infirmary* would benefit by increases in employment, in other words, *would share in the greater prosperity. The employer, if he increased his staff, would be paying his just share on the increased risk due to the greater number employed.* The Board would feel they had done away with the uncertainty and worry of the past and could make this appeal a basis of propaganda, in which practically everyone could join, and through its coming before every employer of labour they in turn would probably bring it to the notice of the merchant classes and thus, indirectly, perhaps influence subscriptions to the infirmary from people who do not give because probably they have never been asked to do so or have not realised their obligations towards their poorer neighbours or the great necessity of doing what they can to help the infirmary to carry on its good work.

I venture to mention that *contributions under this scheme, might, and probably would, be allowed as a general expense by the Inland Revenue Department,* as the money would be used for carrying on the work of the infirmary, whereas if used for a building or extension, it would not be allowed. *Inquiry has been made,* and it is understood this would be allowed to any reasonable amount, which means that, indirectly, the Government are rendering *State aid* towards the expense of carrying on the infirmary *without exercising bureaucratic control over same.*

As propaganda a *list of the firms* or employers joining this scheme could be *published,* say, monthly or quarterly, *without naming the amounts of their subscription,* thus no firm would be able to judge what his neighbours were doing or how many people they employed.

It would have been very gratifying *if the employers' subscription could have been fixed at the equivalent of* 1*d. per week per employee,* equal to the employees' old subscription to the Leeds Workpeople's Hospital Fund, and to which nearly all the employees are now paying 2d. and in some cases 3d. per week, but that would possibly be found too great a tax upon employers, and *that is the reason why I have named half the amount*—that is, 2s. per employee per annum of a ½d. each per week as most likely to meet with universal approval. This policy would surely increase public interest in the infirmary, and widen in all districts general knowledge of its great work, and by that broaden the basis of general subscribers and possibly soon enable the infirmary to deal with a larger number of patients, such as the average 600 to 1,000 always on the waiting list, this being one of the strongest points when putting this scheme before both employers and the general public for their support.

We have italicised the more noteworthy points in this carefully-drawn document, which bears the mark of careful consideration and sound judgment in every line. It remains only to quote the appended table, which explains two important factors in the scheme. The first effect of the table is to make clear at a glance to every firm how much its subscription under the scheme will cost. The second factor explained by the table is the amount by which the Infirmary will benefit from each subscription. The total in each case is not a simple result of arithmetic, because the calculation, though fixed itself by the mere multiplication of the number of employees by 2s., may be gross or not, according to the mood of the Inland Revenue authorities, who may not, for all know their little ways, allow employers to regard it as a

**An Example ef Leadership at Leeds—(*continued*).**

business expense for income-tax purposes. As the table shows, a firm employing fifty men would subscribe £5 a year, and one employing 2,000 £200; but these sums might benefit the Infirmary only to the extent of £3 10s. and £140 respectively if income tax is deducted from them. The table makes this plain at a single glance:—

| | | | Amount Allowed to Fund would be as follows. £ | s. | d. | If Income Tax be deducted. £ | s. | d. |
|---|---|---|---|---|---|---|---|---|
| 50 | employees at | 2s. per year | 5 | 0 | 0 | 3 | 10 | 0 |
| 100 | ,, | 2s. ,, | 10 | 0 | 0 | 7 | 0 | 0 |
| 200 | ,, | 2s. ,, | 20 | 0 | 0 | 14 | 0 | 0 |
| 300 | ,, | 2s. ,, | 30 | 0 | 0 | 21 | 0 | 0 |
| 400 | ,, | 2s. ,, | 40 | 0 | 0 | 28 | 0 | 0 |
| 500 | ,, | 2s. ,, | 50 | 0 | 0 | 35 | 0 | 0 |
| 600 | ,, | 2s. ,, | 60 | 0 | 0 | 42 | 0 | 0 |
| 700 | ,, | 2s. ,, | 70 | 0 | 0 | 49 | 0 | 0 |
| 800 | ,, | 2s. ,, | 80 | 0 | 0 | 56 | 0 | 0 |
| 900 | ,, | 2s. ,, | 90 | 0 | 0 | 63 | 0 | 0 |
| 1,000 | ,, | 2s. ,, | 100 | 0 | 0 | 70 | 0 | 0 |
| 1,250 | ,, | 2s. ,, | 125 | 0 | 0 | 87 | 10 | 0 |
| 1,500 | ,, | 2s. ,, | 150 | 0 | 0 | 105 | 0 | 0 |
| 1,750 | ,, | 2s. ,, | 175 | 0 | 0 | 122 | 0 | 0 |
| 2,000 | ,, | 2s. ,, | 200 | 0 | 0 | 140 | 0 | 0 |

But the explication of a scheme is one thing; its organisation is another. Therefore the experienced men of Leeds have suggested a method of application, which deserves consideration independently and by itself. Once again we prefer to give the text, the *ipsissima verba*, that all the cards may be placed upon the table, that the record may be complete, and that nothing deemed by the authors of this scheme essential to success may be omitted.

### How to Organise Employees' Contributions.

### THE SUGGESTED METHOD AT LEEDS.

That this scheme or fund should be treated as a separate account from the ordinary subscriptions, both in bookkeeping and collection of the money for same.

That the present staff have charge, with any additional assistance required, until the organisation is complete, after which I anticipate the great bulk of the subscribers to the scheme will remit their amount at the end of each period, just as they now do their ordinary accounts.

I would suggest, in order to make this easy for each contributor, that a *special blank statement form (say similar to copy)* be sent by the Treasurer in December and June—the month before payment is due—with space as shown for them to fill in their average number of employees over the period, which at 2s. per head would be so much, which they would then fill in and it would get passed for payment along with their other accounts. *A vast amount of time would be saved over the present system of collection*, and this time could be utilised to widen the basis and increase the number of private subscriptions to the general fund.

I would suggest that it be particularly brought to the notice of each contributor that it would be so *much more helpful to the institution if the subscriptions be paid half-yearly* instead of once a year, as under the old system; the amount would not seem so large if made at twice, and, owing to the shorter interval, every firm would soon begin to look upon these contributions as a part of its establishment charges, and, what is more important, it would make it easier to obtain retrospective payment back to July last.

In working out the suggested method of retrospective payment under the scheme, I would suggest that last July should be taken as the datum line, and that the next full year should date from July next—payable half in that month and the next half in January 1921, so that all the firms who come into the scheme may easily adjust these accounts, if already subscribers, by remitting the exact amount between the payment they have already made and what they would have paid if this scheme had been working in July last.

For example, if a contributor subscribed, say, £10 last September, and his average number of employees was found to be 300, his contribution for the period July 1919 to end of June 1920 at 2s. per head would be £30, less the £10 he had already paid in September last, and he would thus pay into the fund this month the difference of £20, which would clear him until July next, when his half-yearly contribution of £15 would be payable, being half of the £30 on the number of his employees, or more or less according to the actual average number.

The beauty of this arrangement is that *it will help to provide a considerable sum of money at once*, both from old and new subscribers, for urgent needs, and at the same time be *obtained on a business basis from willing givers who are now beginning to understand, by this scheme, the position they are in*, and to feel they know where they are giving, and just how much is their share in doing so, and to know they are not giving more or less than their neighbours, and that when an uncertain visitor calls their consciences are relieved of perhaps many times having to say they *are out* when they *are in*.

Before launching the scheme I wish to suggest that the Chairman of the Leeds Public Dispensary, Mr. W. Penrose Green, who is a warm supporter of the scheme, and if possible a member of the Committee responsible for the Women's and Children's Hospital, Leeds District Nursing Association, and the Maternity Hospital be asked to meet the special Finance Committee, and that *the exact proportion each institution should receive from the fund should be fixed by three prominent citizens independent of the institutes interested*—viz., the Lord Mayor, Mr. Duncan; the Stipendiary Magistrate, Mr. C. N. Atkinson; and the Town Clerk, Sir Robert Fox—after which the amounts could be automatically distributed at definite arranged periods.

At present it would appear the employers' direct contribution to the Leeds Public Dispensary total about £800 per annum, which this scheme would considerably increase. The bulk of the subscriptions from employers of labour to the dispensary are of small amounts, and are not at all in keeping or in proportion to the splendid work done, especially for the very poorest class of the community.

These suggestions are put forward for criticism and improvement, and to encourage ideas to make the scheme as easily workable as possible. T. F. BRAIME.

It is a pleasure to read so lucid a statement, which on the face of it seems to provide for every contingency, and has the advantage of not merely co-ordinating income, but also of co-ordinating the smaller with the larger hospitals in the same city. The workmen, the employers, the general infirmary, the smaller institutions, are all interested. The duties of each are made clear, the responsibility

**An Example of Leadership at Leeds**—(*continued*).

of each is fixed on an equitable basis, and the rights of the sick, the subscribers, and the different institutions are recognised. For these reasons a body of sympathy and understanding is created, and that cannot fail to influence that part of the public not directly mentioned. In the atmosphere of sweet reasonableness thus formed, the desire of each intelligent private citizen can only be not to be left out; consequently, one may expect the example set to spread to the at present amorphous body of private donors and subscribers. The root of the whole scheme is voluntary, as it ought to be; it reposes on good-will, and each part makes its own arrangements intelligently. The contrast of this personal interest with the harassing interference of Government departments, whose interest is competitive, and in that sense opposed to the interest of those whom they control, is too startling to be missed.

